# Measurement of ionized calcium with a point-of-care ionometer during etelcalcetide therapy 

**DOI:** 10.5414/CNP104S03

**Published:** 2025-11-28

**Authors:** Andreja Marn Pernat, Jernej Pajek, Jadranka Buturović-Ponikvar

**Affiliations:** 1 Department of Nephrology, University Medical Center Ljubljana, and; 2 Faculty of Medicine, University of Ljubljana, Ljubljana, Slovenia

**Keywords:** ionized calcium, point-of-care ionometer, etelcalcetide, hemodialysis

## Abstract

Introduction: Hypocalcemia is a common and clinically significant side effect of etelcalcetide therapy. The aim of this study was to evaluate the utility of ionized calcium (iCa) measurements with a point-of-care ionometer compared to albumin-corrected total calcium and to assess the incidence of hypocalcemia in patients receiving etelcalcetide therapy using pre-dialysis iCa values. Materials and methods: This was a phase IV, non-interventional, prospective, single-arm, observational study. A total of 20 chronic hemodialysis patients were included in the study. The iCa concentration was determined before dialysis using a point-of-care ionometer (GEM Premier 3000) at the patient’s bedside. Hypocalcemia was defined by a pre-dialysis iCa concentration of less than 0.90 mmol/L. Results: Pre-dialysis corrected total calcium and iCa decreased over time during treatment with etelcalcetide. A statistically significant linear association was observed between point-of-care iCa and albumin-corrected calcium (r = 0.532, p = 0.019; R^2^ = 0.283). Visual comparisons generally showed parallel behavior, but only a moderate correlation. Of 240 iCa values measured, 3 cases (1.25%) were < 0.90 mmol/L and 20 cases (8.3%) were between 0.90 and 0.96 mmol/L. Conclusion: Our results highlight the value of direct iCa monitoring as a practical and sensitive tool for detecting hypocalcemia and guiding etelcalcetide therapy. Bedside measurement enabled timely dialysate calcium adjustments, preventing clinically significant hypocalcemia and treatment discontinuation. Point-of-care iCa monitoring offers a safer, more responsive strategy for optimizing calcium management in hemodialysis patients.

## Introduction 

Etelcalcetide is a calcimimetic used primarily for the treatment of secondary hyperparathyroidism in patients with end-stage renal disease undergoing hemodialysis [1]. A common and clinically significant adverse effect of etelcalcetide is hypocalcemia, which can lead to life-threatening complications such as QTc prolongation, ventricular arrhythmias, seizures, and worsening heart failure [2]. Even a transient decrease in calcium levels has been associated with increased cardiovascular mortality, independent of the drug itself as shown in a study with cinacalcet [3]. These results emphasize the need for careful monitoring of hypocalcemia induced by calcimimetic therapy. Accurate assessment of serum calcium levels in hemodialysis patients is therefore essential. To date, the evaluation of ionized calcium (iCa) versus corrected total calcium during etelcalcetide treatment has not been studied, and real-world data on the incidence of hypocalcemia based on pre-dialysis iCa measurements during chronic etelcalcetide therapy are lacking. 

The aim of this study was to evaluate the utility of iCa measurements using a point-of-care ionometer compared to albumin-corrected total calcium and to assess the incidence of hypocalcemia in patients receiving etelcalcetide therapy using pre-dialysis iCa values. 

## Materials and methods 

This was an observational, non-interventional, prospective, single-arm phase IV study. The study was approved by the National Medical Ethics Committee of the Republic of Slovenia (approval number: 0120-471/2018/6), and all participants gave written informed consent prior to enrolment. The decision to start treatment with etelcalcetide was made independently of study participation, before patients were approached for inclusion in the study. Inclusion criteria were adult patients with secondary hyperparathyroidism undergoing chronic hemodialysis and starting treatment with etelcalcetide, with or without prior use of cinacalcet. Exclusion criteria included long-term regional citrate anticoagulation, use of other calcium-lowering agents (e.g., bisphosphonates, denosumab), pregnancy or lactation, concurrent participation in another clinical trial, or a medical condition that in the judgement of the investigator, made the patient unsuitable for participation. 

A total of 20 patients from two hemodialysis centers at the University Medical Center Ljubljana were enrolled in the study between January 2020 and March 2021. Patients received etelcalcetide according to the summary of product characteristics (SmPC) and clinical routine [4, 5]. All patients were administered etelcalcetide 3 times a week as a bolus injection into the venous line of the dialysis circuit, immediately before or during the rinse-back after each dialysis session. 

Medical history, concomitant diseases and medications, and laboratory data were collected at the beginning of the study and during the follow-up to 12 months or until the study was discontinued. In the event of premature discontinuation of the study, the reason for discontinuation was recorded and all data available up to this point were included in the analysis. 

The iCa level was measured with a point-of-care ionometer (GEM Premier 3000, Instrumentation Laboratory, Lexington, MA, USA) before the start of treatment, and then before each dialysis session in the first week of treatment. Thereafter, iCa was measured weekly until a maintenance phase was reached, followed by at least monthly monitoring. Other laboratory parameters, including intact parathyroid hormone (iPTH), total calcium, albumin, corrected total calcium were measured in the standard clinical laboratory of the hospital before dialysis in the first week after the start of treatment and at least once a month thereafter, according to clinical routine and not as part of the study protocol. 

The primary endpoints were the iCa value and the corrected total calcium level during chronic treatment with etelcalcetide. They were summarized descriptively and presented graphically to visualize the correlation. The secondary endpoint was the occurrence of hypocalcemia, defined as a pre-dialysis iCa concentration < 0.90 mmol/L. The proportion of patients experiencing hypocalcemia and the total number of hypocalcemic events were calculated and assessed for clinical significance. All adverse drug reactions were monitored and recorded during the study period. 

Descriptive statistics were used to characterize the data set. The mean, standard deviation (SD), median, minimum and maximum values, percentage change, and 95% confidence intervals are given for continuous variables. A two-tailed paired t-test was used to determine statistically significant differences between the means of two variables. For variables measured longitudinally (e.g., iCa, total Ca, iPTH), trends were visualized by plotting the mean. To evaluate the relationship between corrected total calcium and iCa, both correlation using Pearson’s method and linear regression analyses were performed. For categorical variables, the number and percentage of participants in each category are reported. All statistical analyses were performed using JASP software, version 0.16.3 (University of Amsterdam, The Netherlands). 

## Results 

We included 15 men and 5 women with a mean age of 55.9 ± 18.2 years, all undergoing hemodialysis for an average of 5.5 ± 4.3 years. The demographic and clinical characteristics of the study population are summarized in Table 1. Vascular access for hemodialysis was an arteriovenous fistula in 85% of patients, while the remaining 3 patients had jugular central venous catheters. Seven patients had previously received oral cinacalcet in daily doses of 30 mg (n = 2), 60 mg (n = 1), 90 mg (n = 1), 120 mg (n = 1), and 180 mg (n = 2). In all 7 cases, cinacalcet therapy was discontinued for a 1-week washout period. 80% of patients in our cohort were receiving active vitamin D therapy with calcitriol, alfacalcidol, or paricalcitol as part of their standard treatment for secondary hyperparathyroidism during the study period. The starting dose of etelcalcetide was 5 mg (15 mg per week), and in 3 patients the dose was titrated. One patient received an additional 2.5 mg (for a total of 22.5 mg per week), and 2 patients were titrated to 30 mg per week in 5 mg increments (Table 1). 

Only 50% of the patients completed the entire 1-year follow-up period. Among the 10 patients who discontinued the study prematurely, the reasons were as follows: 4 patients underwent kidney transplantation (2 after 3 months, 1 after 7 months and 1 only 1 week after starting therapy), 2 patients were transferred to another dialysis center, 2 patients switched to hemodialysis with long-term regional citrate anticoagulation, 1 patient started denosumab treatment, and 1 patient voluntarily withdrew from the study due to distrust of the drug. 

**Figure 1:**
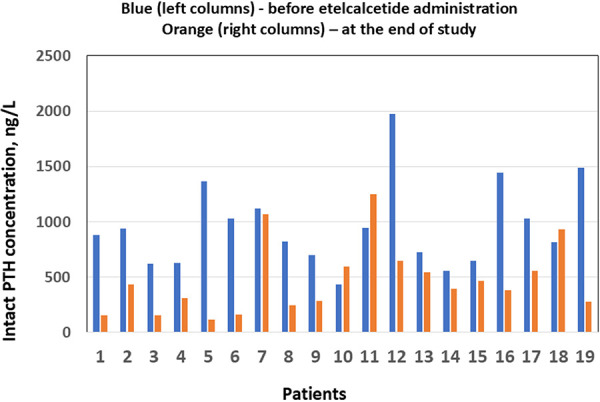
Mean concentrations of intact parathyroid hormone (PTH) prior to dialysis before the first administration of etelcalcetide (left/blue columns) and at the end of the study (right/orange columns) in 19 patients.

**Figure 2:**
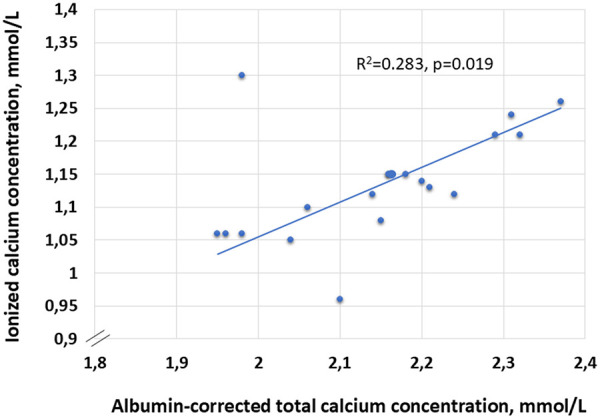
Scater plot comparing pre-dialysis mean albumin-corrected total calcium and ionized calcium concentrations during treatment with etelcalcetide in 19 study patients.

Data from all patients were included in the analysis, except for the patient who underwent a kidney transplantation after receiving only 4 doses of etelcalcetide. Table 2 shows the mean pre-dialysis laboratory parameters for 19 patients at baseline and at the last dialysis session. Etelcalcetide therapy resulted in a highly significant reduction in intact PTH levels in most patients (p = 0.0004; mean change: –44.1%) as shown in Table 2 and Figure 1. Albumin-corrected total calcium levels decreased significantly in the entire cohort (p = 0.02; mean change: –4.5%). Mean iCa was 1.13 mmol/L before treatment and decreased to 1.08 mmol/L during etelcalcetide therapy (mean change: –4.4%). Although this trend indicates a decrease in iCa of 0.05 mmol/L, the difference was not statistically significant (p = 0.125) (Table 2). A visual comparison of iCa and corrected total calcium concentrations prior to dialysis, shown in Figure 2, demonstrated a statistically significant linear association between point-of-care iCa and routine laboratory values of albumin-corrected total calcium in 19 patients receiving etelcalcetide. The correlation coefficient was r = 0.532 (p = 0.019). The coefficient of determination was moderate (R^2^ = 0.283), indicating that ~ 28.3% of the variability in iCa can be explained by corrected calcium. 

Of 240 point-of-care measurements of iCa taken at the bedside, the iCa concentration fell below 0.90 mmol/L 3 times in 2 patients (recorded values: 0.81, 0.84, 0.85 mmol/L). In response, the calcium concentration in the dialysate was increased from 1.50 to 1.75 mmol/L in 1 patient and from 1.25 to 1.50 mmol/L in the other. In addition, 2 measurements showed an iCa value of exactly 0.90 mmol/L and 18 measurements were in the range of 0.91 – 0.96 mmol/L. This corresponds to an incidence of 1.25% for hypocalcemia (iCa < 0.90 mmol/L) and 8.3% for slightly reduced iCa values (0.90 and 0.96 mmol/L). At the beginning of the study, 16 out of 20 patients were dialyzed with a calcium concentration of 1.5 mmol/L. Two patients received a dialysate calcium of 1.75 mmol/L and the remaining 2 of 1.25 mmol/L. By the end of the study, the number of patients using a higher dialysate calcium concentration increased: 2 more patients were switched to 1.75 mmol/L, and 1 patient was switched from 1.25 to 1.50 mmol/L. No episode of hypocalcemia led to discontinuation of etelcalcetide therapy. 

## Discussion

This study provides real-world data on the use of point-of-care monitoring of iCa during chronic treatment with etelcalcetide in hemodialysis patients with secondary hyperparathyroidism. Our results demonstrate the clinical relevance of iCa measurement in detecting early or transient episodes of hypocalcemia that may not be immediately or accurately captured by standard laboratory tests of total or albumin-corrected calcium. 

Accurate assessment of serum calcium is crucial in hemodialysis patients, as hypocalcemia can exacerbate secondary hyperparathyroidism, impair bone mineralization, and has been associated with increased mortality [6]. In circulation, approximately of 40% of calcium is bound to proteins (mainly albumin), 12% is complexed with anions such as phosphate, lactate, citrate, and bicarbonate, while 48% is present as free iCa, the biologically active fraction. Previous studies suggest that iCa is a more physiologically relevant measure, particularly in dialysis patients, where fluctuations in albumin and pH may limit the reliability of corrected total calcium [7, 8]. Nevertheless, the SmPC for Parsabiv, Amgen Inc., Thousand Oaks, CA, USA, (etelcalcetide) recommends using corrected serum calcium to guide therapy [4]. According to the K/DOQI guidelines, total calcium should be adjusted for serum albumin (if < 40 g/L) to better estimate biologically active free calcium [6]. A commonly used correction formula is: corrected calcium (mmol/L) = total calcium (mmol/L) + 0.02 × [40 − serum albumin (g/L)] [6]. However, albumin-corrected total calcium may underestimate hypocalcemia and overestimate hypercalcemia in dialysis patients, potentially leading to suboptimal therapeutic decisions [9, 10]. The importance of iCa monitoring has also been emphasized in relation to cardiovascular risk and arrhythmogenic potential, supporting the superiority of direct iCa measurement over albumin-corrected total calcium in this population [11, 12]. While iCa testing was once limited to specialized laboratories [13], point-of-care ionometers now allow real-time bedside measurement, enabling immediate detection and correction of hypocalcemia, which may help to improve the safety of calcimimetic therapy. To our knowledge, no previous studies have specifically investigated iCa monitoring during etelcalcetide treatment. 

In our cohort, we found a moderate correlation between point-of-care iCa and albumin-corrected total calcium, with corrected calcium explaining less than one-third of the variability in iCa. This only partly confirms their parallel behavior during etelcalcetide therapy and suggests that albumin correction alone does not adequately reflect calcium status and that additional physiological determinants contribute to iCa regulation, particularly in the context of etelcalcetide-induced hypocalcemia. Importantly, while corrected total calcium decreased significantly during treatment, iCa decline was not significant. This discrepancy may reflect prompt clinical interventions in response to the calcium-lowering effect of etelcalcetide, most notably through adjustment of dialysate calcium concentration. At study initiation, most patients were dialyzed with a calcium concentration of 1.50 mmol/L, but by the end of follow-up, a larger proportion were receiving dialysate with increased calcium concentration. The observed increase in pre-dialysis calcium levels following these adjustments further supports the clinical utility of bedside iCa monitoring, which enables timely and targeted interventions that may not be initiated based solely on routine laboratory results. 

Overall, hypocalcemia was rare in our study. Only 1.25% of the 240 point-of-care measurements showed an iCa value < 0.90 mmol/L before dialysis, and 8.3% of the measurements were in the low-normal range (0.90 – 0.96 mmol/L). These findings suggest that point-of-care iCa monitoring was effective in preventing clinically significant hypocalcemia. This approach not only allowed immediate management through dialysate calcium adjustment but also individualized titration of etelcalcetide therapy. Importantly, no patient discontinued therapy due to hypocalcemia, in contrast to previous studies, which reported that at least 1% of patients discontinued calcimimetic therapy due to hypocalcemia, which was usually monitored using corrected total serum calcium [1, 14]. 

Taken together, these findings underline the importance of implementing point-of-care iCa monitoring in hemodialysis units. Such an approach could also be valuable in patients receiving denosumab for osteoporosis, as the combination with etelcalcetide carries a high risk of severe hypocalcemia. Bedside iCa monitoring may therefore represent a safer and more responsive strategy for managing calcium balance in high-risk populations. 

This study has several limitations, including the small sample size, the single-center design, and the lack of a control group. Nevertheless, the prospective design and real-world applicability of the data add to the limited existing evidence on iCa monitoring associated with etelcalcetide therapy. Further studies are warranted to validate these findings in larger patient populations, to establish standardized thresholds for iCa-guided interventions, and to determine the impact of this monitoring approach on clinically meaningful outcomes. 

## Conclusion 

Our study demonstrates that point-of-care monitoring of iCa is a practical and sensitive tool for the early detection of hypocalcemia in hemodialysis patients treated with etelcalcetide. Direct bedside measurement of iCa allows timely adjustments of dialysate calcium concentration, which effectively prevented clinically significant hypocalcemia and treatment discontinuation. These findings support the use of point-of-care iCa monitoring as a strategy to optimize calcium management and enhance the safety of calcimimetic therapy. 

## Acknowledgments 

The authors would like to thank all hemodialysis patients and nurses of the Department of Nephrology of the University Medical Center Ljubljana for their contribution to our study. 

## Authors’ contributions 

J.B.P. conceived and designed the study. A.M.P and J.P. contributed to patient recruitment and data collection. A.M.P. performed the statistical analysis and wrote the manuscript. J.B.P. and J.P. critically revised the manuscript and approved the final version for publication. 

## Funding 

This research was funded by the Slovenian Research and Innovation Agency (research core funding No. P3-0323) and by Amgen GmbH (Investigator Initiated Trial, Study code EteliCa, No. 20187022). 

## Conflict of interest 

J.B.P. served as principal investigator and A.M.P. as investigator for this study. J.B.P., A.M.P., and J.P. have received speaker’s honoraria from Amgen. 

**Table 1.** Demographic and clinical characteristics of the patients and reasons for discontinuation of the study.

Patient No	Gender	Years of age	Primary renal disease	Comorbidities	Dialysis vintage (years)	Type of vascular access	Vitamin D and analogs, daily dose	Dialysate calcium, mmol/L	Prior cinacalcet use, daily dose, mg	Etelcalcetide weekly dose, mg, → dose change	Reason for discontinuation
1	M	62	ADPKD	AH	10	AVF	Calcitriol 0.5 µg, CaCarbonate 0.5 g	1.25	0	15	/
2	M	56	Balkan nephropathy	AH	9	AVF	Calcitriol 0.5 µg, CaCarbonate 1.5 g	1.50	0	15	/
3	M	57	IgA nephropathy	AH	14	AVF	Calcitriol 0,5 µg	1.50	0	15	/
4	F	42	Alport syndrome	/	13	AVF	Paricalcitol 3 µg, CaCarbonate 3 g, Cholecalciferol 15,000 E	1.75	180	15	/
5	M	39	IgA nephropathy	AH	1	AVF	CaCarbonate 3.5 g, Cholecalciferol 10,000 E	1.5 → 1.75	120	15 → 30	/
6	M	61	Malignant hypertension	AH	2	AVF	Cholecalciferol 20,000 E	1.5	0	15	/
7	M	30	Left kidney agenesis, right kidney unknown etiology	AH	1	AVF	Paricalcitol 4 µg, CaCarbonate 2 g, Cholecalciferol 10,000 E	1.5	180	15	Tx after 7 months
8	M	82	ADPKD	AH	1	AVF	Alfacalcidol 0.25 µg, Cholecalciferol 10,000 E	1.5	90	15 → 25	/
9	M	74	IgA nephropathy	AH	2	AVF	Alfacalcidol 3 µg, Cholecalciferol 10,000 E	1.25 → 1.5	30	15 → 30	/
10	M	77	Solitary kidney unknown etiology	AH	7	AVF	CaCarbonate 1 g, Cholecalciferol 10,000 E	1.5	0	15	/
11	M	27	IgA nephropathy	AH	7	AVF	Calcitriol 0.25 µg, CaCarbonate 2 g	1.5	0	15	Dialysis centre transfer after 2 months
12	F	22	Atypical HUS	AH	8	AVF	Alfacalcidol 1 µg, CaCarbonate 1 g	1.5	0	15	Tx after 3 months
13	M	67	Pyonephrosis	AH	2	AVF	Calcitriol 0.25 µg, CaCarbonate 3 g, Cholecalciferol 10,000 E	1.5	0	15	Dialysis centre transfer after 7 months
14	M	63	Multiple myeloma	Right jugular vein thrombosis	1	Two jugular CVC	Calcitriol 0.5 µg, CaCarbonate 1 g	1.5	0	15	Switched to citrate anti-coagulation after 3 months
15	M	32	IgA nephropathy, TMA	AH	2	AVF	Calcitriol 0.25 µg, Cholecalciferol 10,000 E	1.5	0	15	Tx after 3 months
16	F	67	ADPKD	AH	11	AVF	Calcitriol 0.5 µg	1.5	0	15	/
17	F	75	Myoglobinuric AKI precipitated the progression of underlying diabetic nephropathy	Type 2 diabetes, AH, PAOD, chronic pancreatitis	1	Two jugular CVC	Calcitriol 0.25 µg, CaCarbonate 3 g, Cholecalciferol 20,000 E	1.5 → 1,75	0	15	Switched to citrate anti-coagulation after 4 months
18	F	75	Hypertensive nephrosclerosis	AH, CABG	5	Two jugular CVC	CaCarbonate 1 g Cholecalciferol 20,000E	1.5 → 1,75	0	15	Withdrew consent, she did not trust the drug
19	M	61	ANCA vasculitis	Steroid-induced diabetes	7	AVF	Paricalcitol 3 µg CaCarbonate 3 g	1.5	30	15	Tx after 4 dosages of etelcalcetide
20	M	49	IgA nephropathy, kidney cancer	AH	7	AVF	Calcitriol 0.25 µg CaCarbonate 3 g Cholecalciferol 10,000 E	1.75	60	15	Denosumab treatment

ADPKD = autosomal dominant polycystic kidney disease; AKI = acute kidney injury; ANCA = anti-neutrophil cytoplasmic antibody; AH = arterial hypertension; AKI = acute kidney injury; AVF = arteriovenous fistula; CABG = coronary artery bypass graft; CaCarbonate = calcium carbonate; CVC = central venous catheter; F = female; HUS = hemolytic uremic syndrome; M = male; PAOD = peripheral arterial occlusive disease; TMA = thrombotic microangiopathy; Tx = kidney transplant.

**Table 2.** Mean laboratory parameters for 19 patients before and at the end of treatment with etelcalcetide. All analytes were measured before hemodialysis treatment.

Parameter, units	Etelcalcetide	Mean	Range Min – Max	Standard deviation	Median	95% Conf. interval	
						Lower	Upper
Intact parathyroid hormone, ng/L	Before	956	432 – 1,972	385	851	764	1,147
	During treatment	472	118 – 1,248	317	411	314	629
Albumin-corrected calcium, mmol/L	Before	2.15	1.95 – 2.37	0.12	2.15	2.08	2.21
	During treatment	2.05	1.80 – 2.35	0.17	2.06	1.96	2.13
Ionized calcium, mmol/L	Before	1.13	0.96 –1.30	0.08	1.13	1.09	1.17
	During treatment	1.08	0.92 – 1.49	0.14	1.05	1.01	1.15
